# Dr. Henry F. Fullerton

**Published:** 1918-04

**Authors:** 


**Dr. Henry F. Fullerton,** Buffalo University 1875, died in Buffalo Feb. 13, age 84. He was principal of Grammar School No. 16, Buffalo, for nearly fifty years. He never practiced medicine.

